# Prevalence and Determinants of Hyponatremia in Hospitalized Patients at a Tertiary Care Center in Mumbai

**DOI:** 10.7759/cureus.100344

**Published:** 2025-12-29

**Authors:** Anushka Kulkarni, Avinash Jadhao, Anita Chalak

**Affiliations:** 1 Biochemistry, Seth Gordhandas Sunderdas Medical College and King Edward Memorial Hospital, Mumbai, IND; 2 Biochemistry, Government Medical College, Washim, Washim, IND

**Keywords:** determinants, hospitalized patients, hyponatremia, india, prevalence

## Abstract

Background

Hyponatremia is frequently observed as one of the most common electrolyte imbalances in hospitalized patients. Several medical conditions and factors may contribute to the development of hyponatremia, and it is intricately linked with a spectrum of adverse outcomes within clinical settings.

Methodology

This prospective, observational study was designed to assess the prevalence and determinants of hyponatremia in hospitalized patients. Patients aged 18 years or older who were admitted for >24 hours in the ward during the study duration of six months were screened for hyponatremia. In total, 355 patients were studied, of whom four were lost to follow-up.

Results

Our study found the prevalence rate of hyponatremia to be approximately 29.36%. The majority of patients were elderly (>60 years) (215/48.72%) and male (201/58.40%). The most common diagnosis leading to hyponatremia was found to be multifactorial, with an interplay of various disorders, additional comorbidities, and/or drugs such as diuretics. Patients were also commonly diagnosed with liver diseases (48/13.68%), endocrine disorders (47/13.39%), and syndrome of inappropriate antidiuretic hormone secretion (46/13.11%). There was no significant difference in the length of stay among mild, moderate, and severe hyponatremia groups (p = 0.67).

Conclusions

Our study confirms a significant prevalence of hyponatremia in Indian hospital settings. Elderly individuals are most affected, emphasizing the importance of understanding age-related factors. Disease distribution varies by gender, indicating the necessity of tailored approaches. Severity of hyponatremia correlates with mortality risk, emphasizing the need for timely interventions.

## Introduction

Hyponatremia, characterized by a serum sodium ion concentration below 135 mEq/L, is frequently observed as one of the most common electrolyte imbalances in hospitalized patients [1]. The intricate regulation of total body water and electrolyte concentration, orchestrated by mechanisms such as antidiuretic hormone action, renin-angiotensin-aldosterone system, and thirst mechanism, can be disrupted by various factors leading to hyponatremia [2]. Several medical conditions and factors may contribute to the development of hyponatremia. Among these are syndrome of inappropriate antidiuretic hormone secretion (SIADH) [3], congestive heart failure, chronic kidney disease, and the use of diuretics [4,5]. Hyponatremia is intricately linked with a spectrum of adverse outcomes within clinical settings. Its presence heralds not only increased mortality rates but also amplifies morbidity [6], complicating the course of illness for afflicted individuals. Furthermore, the ramifications extend beyond mere health outcomes [7], exerting a tangible impact on healthcare infrastructure and resource allocation.

Moreover, the risk of hyponatremia escalates with advancing age [8], making age an important factor in understanding its prevalence and implications. Studies suggest a wide prevalence range of hyponatremia among Indian hospitalized patients, spanning from 5.2% to 28.8% [9-11]. Despite often being asymptomatic, untreated mild hyponatremia can lead to severe complications, including neurological and gastrointestinal symptoms [12,13]. Chronic and mild hyponatremia can yield deleterious impacts on both the central nervous system and bone health. This condition manifests in various detrimental effects, including gait instability and attention deficits [14].

In clinical settings, hyponatremia can be categorized into admission hyponatremia and hospital-acquired hyponatremia, with the latter comprising a significant portion of cases [15-17], often due to inadequate management practices.

Despite the wealth of literature addressing specific subgroups and medical conditions, a comprehensive understanding of hyponatremia in the Western Indian population remains limited. A study conducted by Jain et al. [9] assessed the clinico-etiological profile of hyponatremia among elderly age group patients in Sikkim. In a study by Babaliche et al. [18] (2017), patients admitted with hyponatremia in the medical intensive care unit (ICU) often present with vomiting followed by confusion as the most common complaint. SIADH emerges as the leading etiology for hyponatremia in this setting. Regional studies, such as those by Chatterjee et al. [19] (2012) in Eastern India and Sood et al. [20] (2020) in the sub-Himalayan region, contribute to a broader understanding of hyponatremia’s clinical profile in diverse geographical areas. However, the above studies have assessed hyponatremia determinants in populations limited by age (elderly), region, or ICU admission. Thus, we undertook a study with a broader population to fill the gap in the current understanding of hyponatremia in India.

Aims and objectives

This study aimed to (1) investigate the prevalence of hyponatremia among hospitalized patients; (2) study the various types of hyponatremia based on severity and assess their determinants; and (3) study the association between determinants of hyponatremia with length of stay and mortality.

## Materials and methods

Study design

This hospital-based, prospective, observational study was conducted at a tertiary care center in Mumbai, India, spanning a six-month period commencing on October 17, 2023, to April 17, 2024.

Population and sample

The prevalence of hyponatremia among Indian patients undergoing hospitalization varies from 5.2% to 28.8% [9-11]. To ensure statistical reliability, the sample size for further investigation was calculated using the single proportion formula. This calculation assumed a hyponatremia prevalence of 30%, chosen as a conservative estimate within the observed range, a confidence level of 95, a marginal error of 5% was deemed acceptable, and a 10% non-response rate was assumed. The resultant final sample size amounted to 355 participants.

Inclusion and exclusion criteria

**Figure 1:**
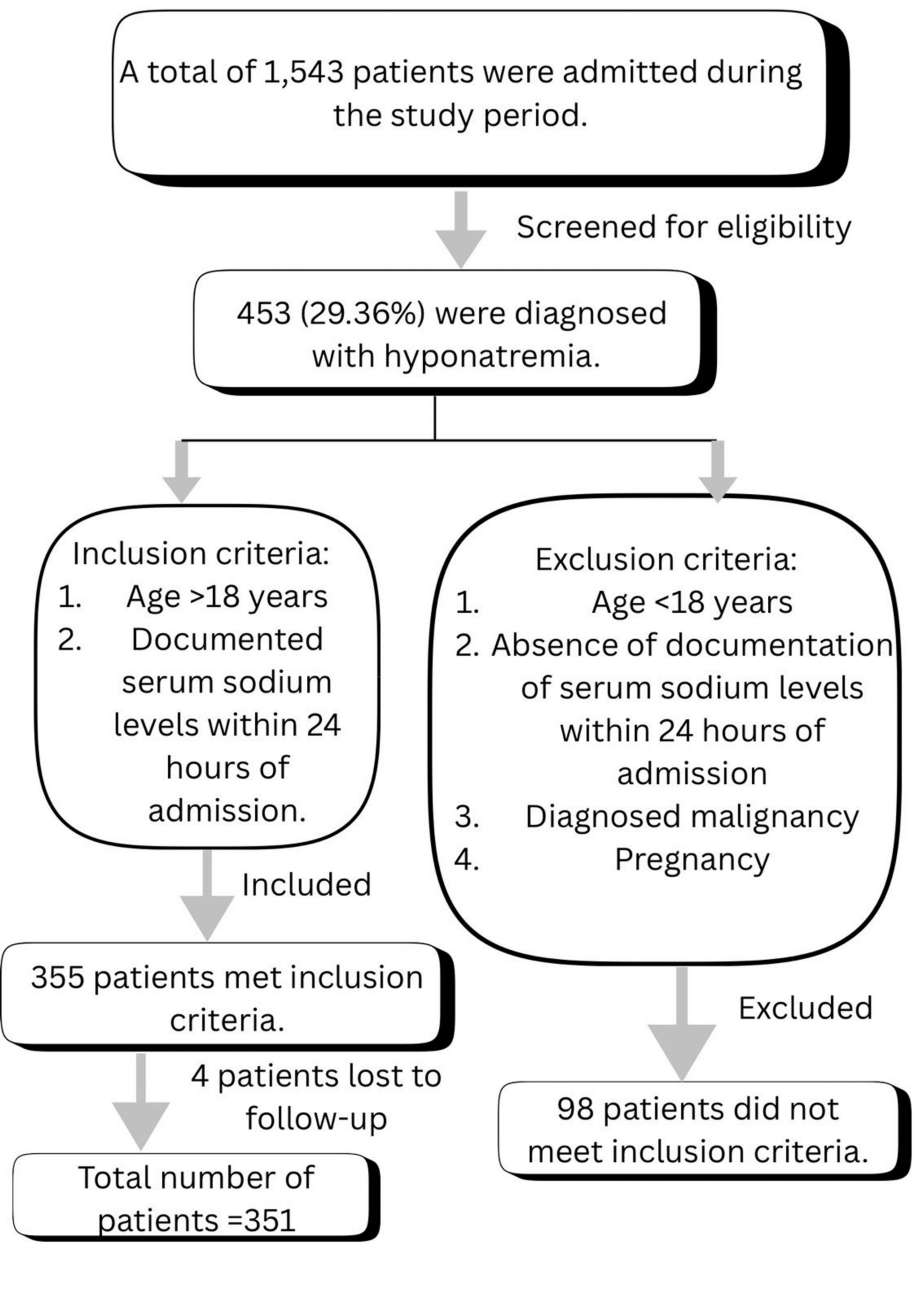
Patient selection process.

Patients aged 18 years or more with serum sodium levels <135 mEq/L on admission or during hospital stay were included in the study. Patients with no serum sodium concentration levels available in the first 24 hours after admission, patients diagnosed with malignancy, and pregnant women were excluded from the study. The patient selection process is illustrated in Figure 1.

Measurement standardization

Serum sodium values were obtained from standardized hospital laboratory assays routinely used for all admitted patients, ensuring uniform measurement across the study population.

Data collection

Information was extracted manually from patients’ records and entered into Microsoft Excel version 2019 (Microsoft Corp., Redmond, WA, USA). Procedures ensured data accuracy and completeness.

Study variables

Independent variables included age, gender, and main diagnosis at discharge, while dependent variables comprised serum sodium levels on admission and during hospital stay, length of stay, and mortality.

Age groups were delineated into three categories: young adults, encompassing individuals aged between 18 and 45 years; middle-aged, representing those falling within the 46 to 60 years bracket; and elderly, denoting individuals surpassing 60 years of age. Hyponatremia was categorized according to severity as mild (sodium ion concentration = 130-135 mEq/L), moderate (sodium ion concentration = 125-129 mEq/L), or severe (sodium ion concentration <125 mEq/L).

Recovery was defined as correction of sodium ≥135 mEq/L with clinical improvement and discharge alive. Deaths included patients who passed away in the hospital without correction of sodium levels.

Statistical analysis

Statistical analysis was performed using GraphPad Prism version 10. Descriptive statistics were employed to scrutinize sociodemographic particulars and other independent variables, delineating results through frequencies and percentages. Chi-square tests were used to discern associations between dependent and independent variables, with statistical significance established at p-values <0.05. Furthermore, a one-way analysis of variance (ANOVA) test was employed to juxtapose the length of stay among patients exhibiting varying degrees of hyponatremia. Owing to the study design, limited number of outcome events, and sample size within severity subgroups, multivariable or risk-adjusted analyses were not performed, and causal associations between hyponatremia severity and outcomes cannot be established.

Ethical considerations

Before enrolment, patients and their relatives were provided with comprehensive information, and consent was obtained in the local language, ensuring their informed participation. Additionally, stringent measures were implemented to safeguard patient confidentiality throughout the duration of the study. This study was performed in accordance with the Helsinki Declaration of 1975 and after obtained due approval from the Institutional Ethics Committee (approval number: EC/OA-143/2023).

## Results

During the six-month study period, a total of 1,543 patients were admitted to the Medicine ward. Among them, 453 patients were diagnosed with hyponatremia, indicating a prevalence rate of approximately 29.36%. This statistic underscores the notable frequency of this electrolyte imbalance within the patient cohort during the investigation.

Furthermore, within the study’s scope, 355 out of the 453 patients (78.36%) identified with hyponatremia were enrolled. Unfortunately, four individuals were lost to follow-up during the prospective phase of the study, resulting in a final participant count of 351 individuals.

Table 1 shows the distribution across age groups, which indicates that the majority of participants were elderly (>60 years), comprising 61.25% (215) of the total sample. The gender distribution shows that there were more male participants (201/57.26%) than female participants (150/42.73%). Overall, 245 patients had admission hyponatremia, i.e., hyponatremia within 24 hours of admission (69.8%).

**Table 1:** Demographic distribution of study participants.

Demographic	Number of patients	%
Age		
Young adults (18 to 45 years)	48	13.67%
Middle-aged (46 to 60 years)	88	25.07%
Elderly (>60 years)	215	61.25%
Gender		
Male	201	57.26%
Female	150	42.73%
Hyponatremia type		
Admission hyponatremia	245	69.8%
Hospital-acquired hyponatremia	106	30.2%
Total	351	100%

Table 2 shows that the highest incidence among male patients was liver disease at 19.02% (39), while for female patients, it was SIADH at 20.55% (30). Overall, the most common diagnosis was drug-induced conditions, accounting for 17.38% (61) of all cases. Drug-induced hyponatremia may be caused by thiazide diuretics, loop diuretics, selective serotonin reuptake inhibitors, antiepileptics (carbamazepine, oxcarbazepine), antipsychotics, angiotensin-converting enzyme inhibitors, angiotensin receptor blockers, proton pump inhibitors, via SIADH or renal sodium loss. Table 3 provides valuable insights into the prevalence of these conditions among the patient population.

**Table 2:** Distribution of participants according to diagnosis. SIADH: syndrome of inappropriate antidiuretic hormone secretion

Diagnosis	Male patients	Male patients %	Female patients	Female patients %	Total	Total Percentage
Cardiovascular disease	11	5.37%	8	5.48%	19	5.41%
Multifactorial	24	11.71%	7	4.79%	31	8.83%
Endocrine disorder	33	16.10%	14	9.59%	47	13.39%
Liver disease	39	19.02%	9	6.16%	48	13.68%
Cerebrovascular accident	11	5.37%	10	6.85%	21	5.98%
Neurological disease	5	2.44%	4	2.74%	9	2.56%
Pulmonary disease	6	2.93%	7	4.79%	13	3.70%
Kidney disease	13	6.34%	17	11.64%	30	8.55%
Sepsis	15	7.32%	11	7.53%	26	7.41%
Drug-induced	32	15.61%	29	19.86%	61	17.38%
SIADH	16	7.80%	30	20.55%	46	13.11%
Total	205	100%	146	100%	351	100%

**Table 3:** Patient characteristics based on the severity of hyponatremia. Mild hyponatremia: sodium ion concentration = 130-135 mEq/L. Moderate hyponatremia: sodium ion concentration = 125-129 mEq/L. Severe hyponatremia: sodium ion concentration <125 mEq/L. SIADH: syndrome of inappropriate antidiuretic hormone secretion

		Mild hyponatremia	Mild %	Moderate hyponatremia	Moderate %	Severe hyponatremia	Severe %	Total	Total %
Gender	Male	34	50.74%	65	53.71%	102	62.57%	201	57.26%
	Female	33	49.25%	56	46.48%	61	37.42%	150	42.73%
	Total	67	100	121	100	163	100	351	Total
Diagnosis	Cardiovascular	6	8.9%	5	4.13%	8	4.90%	19	5.41%
	Multifactorial	6	8.9%	10	8.26%	15	9.2%	31	8.83%
	Endocrine disorder	11	16.41%	16	13.22%	20	12.26%	47	13.39%
	Liver disease	5	7.46%	20	16.52%	23	14.11%	48	13.67%
	Cerebrovascular accident	3	4.47%	6	4.95%	12	7.36%	21	5.98%
	Neurological disease	1	1.49%	3	2.47%	5	3.06%	9	2.56%
	Pulmonary disease	3	4.47%	4	3.30%	6	3.68%	13	3.70%
	Kidney disease	6	8.95%	12	9.91%	12	7.36%	30	8.54%
	Sepsis	6	8.95%	10	8.26%	10	6.13%	26	7.40%
	Drug induced	12	17.91%	20	16.52%	29	17.79%	61	17.37%
	SIADH	8	11.94%	15	12.39%	23	14.11%	46	13.10%
	Total	67	100 %	121	100%	163	100%	351	100%

**Figure 2:**
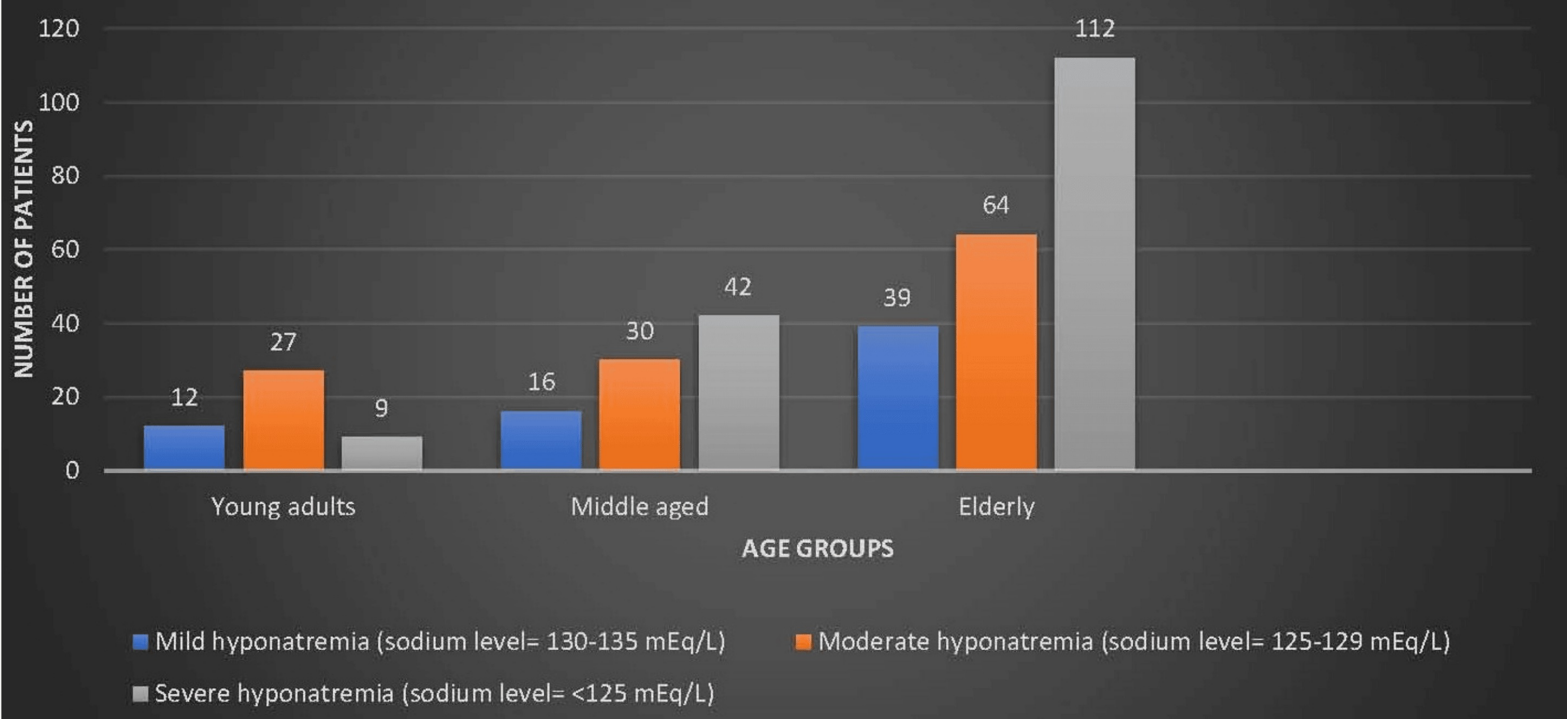
Age-wise distribution of severity of hyponatremia.

Figure 2 demonstrates that in the young adult cohort, aged between 18 and 45 years, there was a discernible shift in the distribution of hyponatremia severity. Specifically, among this demographic subset, only nine (18.75%) patients presented with severe hyponatremia. Among the elderly, aged over 60 years, 112 (52.09%) suffered from severe hyponatremia.

Table 3 shows that male patients made up a vast majority of patients suffering from severe hyponatremia. Among mild hyponatremia cases, drug-induced and endocrine diseases contributed to the majority. Endocrine disorders included hypothyroidism, adrenal insufficiency, uncontrolled diabetes mellitus, and pituitary disorders. Liver disease and drug-induced represented the highest percentage of moderate hyponatremia, followed by endocrine disorders and multifactorial diagnoses. Drug-induced hyponatremia and SIADH were the leading causes of severe hyponatremia.

**Figure 3:**
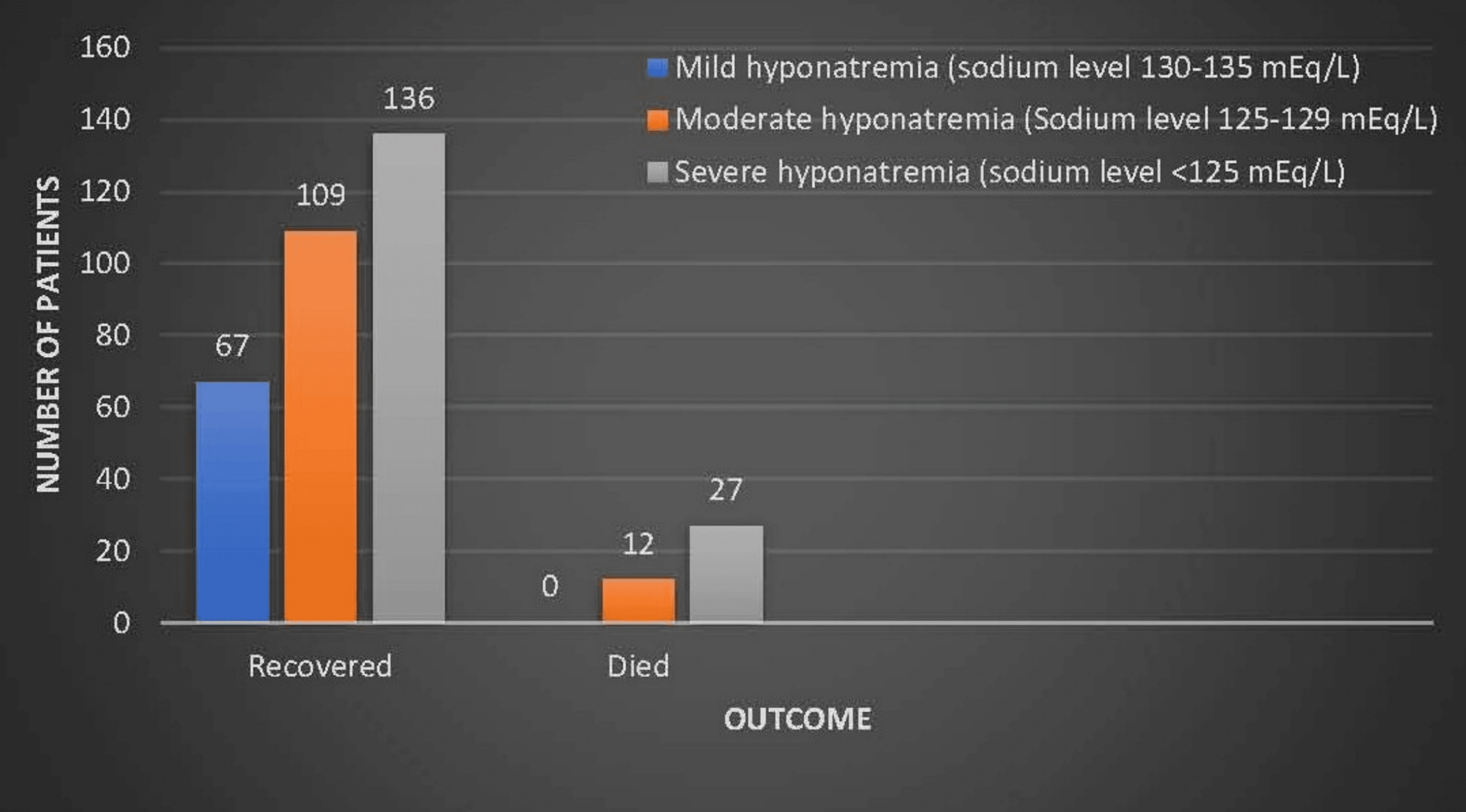
Outcomes of patients admitted with different grades of severity of hyponatremia.

Figure 3 shows that out of 351 patients across all severity levels, 312 patients recovered, making up 88.89% of the total cases. Overall, 39 patients across all severity levels passed away, representing 11.11% mortality in the entire cohort.

The findings presented in Table 4 indicate that there was no discernible disparity in length of stay across the categories of mild, moderate, and severe hyponatremia groups.

**Table 4:** Comparison between length of stay among patients with different degrees of hyponatremia. Mild hyponatremia: sodium ion concentration = 130-135 mEq/L. Moderate hyponatremia: sodium ion concentration = 125-129 mEq/L. Severe hyponatremia: sodium ion concentration <125 mEq/L.

	Mild hyponatremia	Moderate hyponatremia	Severe hyponatremia	P-value
Length of stay in days (mean ± standard deviation)	11.56 ± 9.12	12.46 ± 8.16	12.96 ± 8.26	0.67

## Discussion

The prevalence rate of hyponatremia observed in our study, which stands at 29.36%, closely correlates with previous findings documented in Indian hospital settings. These studies have reported the prevalence of hyponatremia to vary within a range of 5.2% to 28.8% [9-11]. This consistency in prevalence rates across studies underscores the significance of hyponatremia as a common electrolyte imbalance among hospitalized patients in India.

Age distribution

The distribution across these age groups revealed that the majority of participants were elderly individuals (>60 years), comprising 61.25% (215) of the total sample. This distribution is consistent with findings from previous studies. Prior research [15] reported a similar trend in age distribution among hyponatremia patients in India. Understanding the predominance of elderly individuals among hyponatremia patients is essential, as aging is associated with physiological changes that can predispose individuals to electrolyte imbalances. Hyponatremia in the elderly population is influenced by a myriad of factors, including diminished renal function and perturbations in thirst perception [8,15].

Gender distribution

Our study unveiled a notable gender disparity, with a greater proportion of male patients (201/57.26%). This observation resonates with previous research findings, which have consistently shown a higher prevalence of hyponatremia among males. Babaliche et al. [18] documented a comparable gender distribution in their study, where 59% of participants were male, and 41% were female. Similarly, Rahil et al. [21] documented a gender ratio of 62.3% male to 37.7% female among hyponatremia patients. This trend is further supported by a study reported by Chatterjee et al [19]. Similarly, in the study of Sood et al. [20], the ratio of males to females was recorded as 1.25:1.

Classification of hyponatremia

In our study, we categorized the types of hyponatremia observed into two distinct groups: admission hyponatremia and hospital-acquired hyponatremia. The majority of participants in our study, comprising 245 out of 351 individuals (69.8%), were diagnosed with admission hyponatremia. This underscores the importance of recognizing and addressing pre-existing electrolyte imbalances among patients upon hospital admission. The presence of hyponatremia at admission may signify underlying chronic conditions, such as heart failure or renal dysfunction, electrolyte disturbances, or medication use that necessitate careful management throughout the hospital stay.

Moreover, 106 (30.2%) participants in our study were diagnosed with hospital-acquired hyponatremia. These individuals developed hyponatremia during their hospitalization, suggesting potential iatrogenic factors or hospital-related interventions contributing to the onset of hyponatremia. Hospital-acquired hyponatremia highlights the need for vigilant monitoring of electrolyte levels, judicious use of intravenous fluids, and appropriate management of underlying conditions to prevent adverse outcomes associated with hyponatremia in hospitalized patients [2].

Disease distribution

Drug-induced conditions emerge as the most common diagnosis, accounting for 17.38% (61) of all hyponatremia cases. This underscores the importance of medication review and monitoring for adverse drug reactions, particularly in patients presenting with electrolyte abnormalities. This finding of the study correlates with Clayton et al. [22] and Singh et al. [17], underscoring the significant role of diuretics, especially thiazide diuretics, in the onset of hyponatremia.

Similarly, studies by Babaliche et al. [18] and Pillai et al. [10] have identified SIADH as a common cause of hyponatremia in patients admitted to the ICU. These findings stress the importance of considering SIADH in the differential diagnosis of hyponatremia and underscore the significance of implementing appropriate management strategies specific to the underlying cause.

Outcomes by hyponatremia severity

Prior studies conducted by Gill et al. [7] and Stern et al. [23] suggested that hyponatremia severity alone may not be the primary determinant of mortality risk in affected patients. Instead, other factors such as underlying comorbidities, complications, and timely interventions likely play crucial roles in determining patient outcomes. Erasmus and Matsha [24] noted an elevated mortality rate correlating with the severity of hyponatremia.

In our study, although the severe hyponatremia group showed a numerically lower recovery rate and a higher proportion of deaths, our statistical analysis did not assess correlation, and therefore, no association between severity and mortality can be established. These are descriptive trends only. Prior studies underscore the intricate nature of this issue and emphasize the necessity for additional investigation to clarify the underlying mechanisms influencing mortality risk among hyponatremia patients.

Length of stay

The average length of hospital stay remained consistent across all severity levels of hyponatremia, with no notable differences observed. Patients, on average, were hospitalized for approximately 12 to 13 days, irrespective of the severity of hyponatremia. This finding of our study correlates with a study conducted by Baser et al. [25]. These findings suggest that while hyponatremia severity may influence patient outcomes, it does not appear to have a substantial impact on the duration of hospitalization.

Study limitations

Small Sample Size

One notable limitation inherent in this study pertains to its relatively diminutive sample size, a factor that may inadvertently circumscribe the extrapolation of its findings to encompass wider demographic cohorts. Utilizing a larger sample size would offer a more comprehensive understanding of both the prevalence and outcomes associated with hyponatremia.

Short-Term Focus

The predominant focus of the study lies in the assessment of immediate impacts on mortality, thereby potentially overlooking the consideration of long-term outcomes such as readmission rates and prolonged mortality. Consequently, it may fail to comprehensively capture the enduring effects of hyponatremia on patient health over an extended period.

Single-Center Study

A single-center study limits the generalizability of findings because patient populations, clinical practices, and diagnostic criteria may differ across institutions. Additionally, center-specific protocols can introduce systematic bias.

Tertiary Care Center Population

The study population comprises patients seeking medical attention at a tertiary care center. This demographic might not accurately reflect the broader populace, given that individuals accessing care at such facilities could possess distinct characteristics or differing levels of healthcare accessibility compared to the general population.

Study Design Limitation

Owing to the study design, limited number of outcome events, and sample size within severity subgroups, multivariable or risk-adjusted analyses were not performed, and causal associations between hyponatremia severity and outcomes cannot be established. Observed differences in recovery and mortality are therefore descriptive only.

## Conclusions

Our study confirms a significant prevalence of hyponatremia in Indian hospital settings, echoing previous research. Elderly individuals are most affected, emphasizing the importance of understanding age-related factors. Gender differences exist, with males showing a higher prevalence. Classification into admission and hospital-acquired hyponatremia aids in understanding its dynamics. Disease distribution varies by gender, indicating the necessity of tailored approaches. Mortality in hyponatremia patients emphasizes the need for timely interventions. Despite study limitations, our findings stress the importance of individualized patient care and call for further research to address gaps in understanding and improve clinical practices.
